# Multiplexed Proteomic Analysis for Diagnosis and Screening of Five Primary Immunodeficiency Disorders From Dried Blood Spots

**DOI:** 10.3389/fimmu.2020.00464

**Published:** 2020-04-01

**Authors:** Christopher J. Collins, Fan Yi, Remwilyn Dayuha, Jeffrey R. Whiteaker, Hans D. Ochs, Alexandra Freeman, Helen C. Su, Amanda G. Paulovich, Gesmar R. S. Segundo, Troy Torgerson, Si Houn Hahn

**Affiliations:** ^1^Seattle Children's Research Institute, Seattle, WA, United States; ^2^Fred Hutchinson Cancer Research Center, Seattle, WA, United States; ^3^University of Washington School of Medicine, Seattle, WA, United States; ^4^National Institute of Allergy and Infectious Diseases, NIH, Bethesda, MD, United States; ^5^Department of Pediatrics, Universidade Federal de Uberlândia, Uberlândia, Brazil

**Keywords:** primary immunodeficiency disorders, newborn screening, proteomics, immuno-SRM, dried blood spots

## Abstract

Early detection of Primary Immunodeficiencies Disorders (PIDDs) is of paramount importance for effective treatment and disease management. Many PIDDs would be strong candidates for newborn screening (NBS) if robust screening methods could identify patients from dried blood spots (DBS) during the neonatal period. As majority of congenital PIDDs result in the reduction or absence of specific proteins, direct quantification of these target proteins represents an attractive potential screening tool. Unfortunately, detection is often limited by the extremely low protein concentrations in blood cells and limited blood volume present in DBS. We have recently developed a robust novel method for quantification of low abundance proteins in DBS for PIDDs using peptide immunoaffinity enrichment coupled to selected reaction monitoring (immuno-SRM). Here, we further generated a multiplexed Immuno-SRM panel for simultaneous screening of eight signature peptides representing five PIDD-specific and two cell-type specific proteins from DBS. In samples from 28 PIDD patients including two carriers, representing X-Linked Agammaglobulinemia (XLA), Wiskott-Aldrich Syndrome (WAS), X-Linked Chronic Granulomatous Disease (XL-CGD), DOCK8 Deficiency and ADA deficiency, peptides representing each disease are significantly reduced relative to normal controls and patient identification had excellent agreement with clinical and molecular diagnosis. Also included in the multiplex panel are cell specific markers for platelets (CD42) and Natural Killer Cells (CD56). In patients with WAS, CD42 levels were found to be significantly reduced consistent with characteristic thrombocytopenia. A patient with WAS analyzed before and after bone marrow transplant showed normalized WAS protein and platelet CD42 after treatment highlighting the ability of immuno-SRM to monitor the effects of PIDD treatment. The assay was readily reproduced in two separate laboratories with similar analytical performance and complete agreement in patient diagnosis demonstrating the effective standardized methods. A high-throughput Immuno-SRM method screens PIDD-specific peptides in a 2.5-min runtime meeting high volume NBS workflow requirements was also demonstrated in this report. This high-throughput method returned identical results to the standard Immuno-SRM PIDD panel. Immuno-SRM peptide analysis represents a robust potential clinical diagnostic for identifying and studying PIDD patients from easily collected and shipped DBS and supports a significant potential for early PIDD diagnosis through newborn screening.

## Introduction

Primary Immunodeficiency Disorders (PIDD), also referred to as inborn errors of immunity (IEI), are a group of more than 416 rare genetic disorders in which components of the immune system are missing or improperly functioning. Although individually rare, the combined incidence of PIDDs is estimated to be about 1 in 1,200 (1–3). Once diagnosed and treated appropriately, patients can often lead relatively normal lives (4, 5). Curative therapies are also possible, depending on the disorder, with hematopoietic stem cell transplantation (HSCT), enzyme replacement therapy (ERT), or gene therapy (5–11). Almost ubiquitously, early detection of PIDDs is extremely important in controlling and preventing potentially life-threatening infections and chronic sequelae (12, 13).

Early intervention is limited by the difficulty in diagnosing PIDDs clinically and the lack of straightforward population screening tools. Laboratory evaluations are typically elicited by evidence of recurrent and/or chronic infections. After clinical evaluation, laboratory tests that are required for a diagnostic confirmation often involve technically demanding analyses, including immune cell subset analysis, protein expression, and/or enzymatic activity in patient's white blood cells (14). Currently it is not possible to perform these clinical diagnostics from dried blood spots (DBS) as all the tests require either whole blood or isolated peripheral blood mononuclear cell (PBMC) samples. Genetic sequencing is most often used as a final confirmation (14).

An operationally simple mass spectrometry assay using a less invasively collected sample by heel stick would allow for rapid screening of suspected PIDDs. The sensitivity and specificity of tandem mass spectrometry (MS/MS) based proteomic assay can be utilized to reliably measure extremely low abundance peptides in DBS extracts and thus quantify the proteins they represent (15, 16). Furthermore, an assay capable of detecting PIDD patients using DBS would be applicable in newborn screening (NBS) and allow for patient identification before the onset of potentially fatal infections. Newborn screening for T-Cell receptor excision circles (TRECs) and Kappa-deleting element recombination circles (KRECs), DNA fragments released during T-cell and B-Cell maturation respectively, from DBS does exist for severe combined immunodeficiency (SCID) and some forms of agammaglobulinemia. Recently, all US states have implemented TREC screening for SCID, however, a second tier test able to be run from DBS would be beneficial to reduce the false positive rate (17, 18). In addition, TREC/KREC analysis has the potential to miss several SCID subtypes (e.g., ADA, ZAP70, and MHC deficiency) and NBS for other PIDDs does not currently exist due to the lack of screening methods (17, 19–22). Nevertheless, recent advances have demonstrated the ability to screen metabolites related to Adenosine Deaminase (ADA) deficient SCID and purine nucleoside phosphorylase deficiency, and epigenetic markers for immune cell profiles (23–25).

We have previously shown that an MS-based approach for the quantification of signature peptides for BTK, WASP, and a T-Cell marker CD3ε from tryptic digests of PBMCs can be used to screen X-linked agammaglobulinemia (XLA), Wiskott-Aldrich Syndrome (WAS), and SCID, respectively (26). CD3ε was chosen as a general representation of T-Cell number as all SCID patients share T-Cell lymphopenia despite genetic heterogeneity. Each patient in the blinded study was deficient in the signature peptide specific for their respective disease [i.e., XLA patient lacking Bruton's Tyrosine Kinase (BTK) and WAS patient missing WAS protein (WASP), etc.]. These efforts were subsequently extended to include peptide immuno-affinity enrichment coupled to selected reaction monitoring (Immuno-SRM) technology (15, 27), also referred to as Stable Isotope Standards and Capture by Anti-Peptide Antibodies (SISCAPA). Immuno-affinity enrichment of signature peptide biomarkers using anti-peptide antibodies isolates peptides of interest from complex biological matrices. This simplifies the sample matrix, reduces background, and concentrates analytes to enhance the sensitivity of the liquid chromatography-tandem mass spectrometry (LC-MS/MS) assay (28, 29). Immuno-SRM allows for quantification of proteins present at low picomolar concentrations in blood with high reproducibility (30–34). This technique is highly reproducible, multiplexable, and transferrable across laboratories (34–38). Using this methodology in a blinded screen of 82 samples (42 patient samples with 40 normal controls), every molecularly confirmed case of XLA (*n* = 26), WAS (*n* = 11) and 2 out of 3 cases of SCID were significantly reduced in their respective peptides and diagnostic cutoffs allowed for their positive identification (15).

In this study, we expanded the multiplexed proteomic diagnostic panel to screen DBS for five molecularly defined PIDDs using eight signature peptide biomarkers. The current set of targeted PIDDs includes ADA deficiency (ADA) (39, 40), Dedicator of cytokinesis 8 (DOCK8) deficiency (6, 41), X-Linked Chronic Granulomatous disease (XL-CGD) (42, 43), Wiskott-Aldrich Syndrome (WAS) (44, 45), and X-Linked Agammaglobulinemia (XLA) (46, 47). These specific PIDDs were chosen because they are strong candidates for newborn screening when accounting for the Wilson and Jungner criteria for selecting candidate diseases (48). These disorders are well-studied with a good understanding of clinical course, have effective potential treatment, and are relatively frequent. In these cases, however, robust newborn screening methods that can be performed from DBS do not exist. This means that there can be an extended time to diagnosis, resulting in significant negative sequalae. Signature peptides for these conditions serve as primary markers for direct diagnosis of a specific PIDD. Analysis of secondary protein markers such as neural cell adhesion molecule (CD56) and glycoprotein Ib (CD42) provide support for specific diagnoses by generating information as to the counts of Natural Killer (NK) cells and platelets, respectively. Together, eight peptide biomarkers associated with the above conditions are quantified in a multiplex assay simultaneously.

The assay uses custom monoclonal anti-peptide monoclonal antibodies (mAbs) to signature peptides to provide high specificity, reproducibility, and batch-to-batch consistency relative to polyclonal antibodies (pAbs). Normal control ranges were established and blinded sample sets were used to demonstrate the ability to differentiate patients from controls while secondary markers have provided information about the resulting effects on the hematopoietic and immune system. Finally, the multiplexed methods were validated in a second laboratory to show they are highly transferrable and suitable for both diagnostic analysis and high-throughput (HT) NBS.

## Materials and Methods

### Dried Blood Spot Samples

This protocol was approved by the institutional review board of Seattle Children's Hospital (SCH). All subjects gave written informed consent in accordance with the Declaration of Helsinki. Normal control blood samples were purchased from BioIVT (Hicksville, NY). Patient samples were provided by the Seattle Children's Immunology Diagnostic Laboratory including samples that originated at the Universidade Federal de Uberlândia (Uberlândia, Brazil). Additional patient samples were collected at the National Institutes of Health (Bethesda, MD) after written informed consent was obtained on an NIAID-approved research protocol and were sent via air mail. Samples were prepared either by fingerstick or by pipetting 70 μL of blood (per 12 mm spot) onto filter paper cards (Protein Saver 903, Piscataway, NJ). The samples were then dried overnight at room temperature, shipped to SCH, and stored at −80°C until use. DBS samples were analyzed from 175 normal controls. In total, DBS from 29 PIDD patients and carrier samples were analyzed including samples from 7 WAS patient (one before and after HSCT), 11 XLA patient and 2 carrier, 1 DOCK8 deficient patient, 3 XL-CGD patients, 1 AR-CGD patient, and 3 ADA deficient patients.

### Immuno-SRM Reagents

Triton X-100 (T9284-100 mL) and Ammonium bicarbonate (Ambic) (A6141-25G) was purchased from Sigma Aldrich. TPCK-treated Worthington Trypsin (LS003740) was purchased from Worthington. (3-[3-cholamidopropyl = dimethylammonio]-1-propanesulfonate) (CHAPS) (no. 28300), Acetonitrile (ACN) (no. A955, Optima LC/MS grade), Acetic Acid (AA) (no. A11350, Optima LC/MS grade), water (no. W6, Optima LC/MS grade), formic acid (FA) (no. A117, Optima LC/MS grade), phosphate-buffered saline (1 × PBS, no. 10010-023), Dithiothreitol (DTT) (no. 20290) and 1M Tris(hydroxymethyl)aminomethane pH 8 (TRIS) (no. 15568-025) buffer were obtained from Fisher Scientific (Waltham, MA).

Isotope-labeled internal standard (IS) peptides were purchased from either Atlantic Peptides (Lewisburg, PA) or Life Technologies Corporation (Carlsbad, CA). IS peptides were purified to >95% and synthesized to incorporate a heavy stable isotope-labeled C-terminal lysine or arginine. The labeling (^13^C or ^15^N), results in a mass shift of +8 (Lys) or +10 (Arg) Daltons (Da). Aliquots were stored in 5% ACN/0.1% FA at −80°C until use.

PIDD internal standard (IS) peptides were stored as 500× mixtures in 1× PBS + 15% ACN + 0.1% FA + 0.03% CHAPS in H_2_O and diluted to 1X immediately before use. Final peptide concentration in the stock mix and peptide capture experiment are shown in Table S1.

### Selection of Signature Peptides and Antibody Production

Candidate signature peptide selection was done according to published CPTAC guidelines (33). In brief, candidates were generated by *in silico* digestion of proteins to generate tryptic peptides. These sequences were segregated based on length and hydrophobicity. Peptides with potential missed cleavages, methionine, and known post-translational modifications were excluded. Final candidate peptides were screened for uniqueness in the proteome using BLAST tools. If multiple candidate peptides existed, final peptides were selected based on MS response. Final peptide selections for antibody production were made by comparing MS response at equivalent concentrations. Fragmentation patterns for BTK 407 and WASP 274 have been previously reported (26). Fragmentation patterns for all remaining peptides are presented as Figure S1.

Antibody production was performed by Excel Biopharm (San Francisco, CA) and Fred Hutchinson Cancer Research Center Antibody Production Core (FHCRC-APC). At Excel Biopharm, peptide sequences were synthesized with an N-terminal cysteine and conjugated to adjuvant proteins before rabbit immunization. PBMCs from animals with high titers and activity were isolated and then B-Cells were cultured. Responding isolated B-Cells then had their cDNA cloned and antibodies expressed in a mammalian expression system for further screening. Antibodies were tested after B-Cell culture and antibody clone expression using ELISA and Immuno-SRM methods to determine suitability for monoclonal development.

At FHCRC-APC (Seattle, WA), peptide synthesis and adjuvant conjugation were conducted as above before immunization of mice. B-cells from responding animals were isolated and hybridoma cell lines produced. Antibodies were tested at multiple stages in crude bleeds and after hybridoma development using ELISA and Immuno-SRM methods to determine suitability for monoclonal development.

### Antibody Bead Reagent Production

Monoclonal Antibody (mAb) beads were produced by incubating with Protein G coated magnetic beads (Dynabeads, no. 10004D) from Invitrogen (Carlsbad, CA), which were initially washed with 1× PBS + 0.03% CHAPS and magnetic isolation (Millipore, no. 20-4000) (Temecula, CA) of the beads to remove the storage buffer. Beads were mixed by pipetting and subsequently isolated by the magnet. This washing procedure was repeated for a total of three times. Finally, appropriate amount of mAb stock solution was added to the isolated beads at a ratio of 1:2.5 μg mAb:μL bead. Then the antibodies and beads were tumbled overnight at 4°C for coupling. Finally, the immobilized mAb-linked beads were washed twice with 1× PBS + 0.03% CHAPS and resuspended at a concentration of 0.4 μg/μL.

### DBS Extraction, Trypsin Digestion, and Immunoaffinity Enrichment

Protein extraction and tryptic digestion were performed with a modified procedure to those previous reported (15). Two 6.35 mm diameter punches were taken from the DBS cards and placed into 96-well plates (MASTERBLOCK, Greinier, no. 786201) from Thermo Scientific (Chicago, IL) covered with adhesive seal (ThermalSeal, no. 12-168) from Genesee Scientific (San Diego, CA) during the incubation process. These punches were submerged in 300 μL of 0.1% Triton X-100 in 50 mM ambic buffer. Dithiothreitol (DTT) was then added at a concentration of 0.2M DTT in H_2_O. After vortex, protein extraction proceeded for 30 min at 37°C with agitation. After protein extraction, 60 μg of Trypsin was added and incubated for 2 h at 37°C with agitation. The resulting peptide mixture was directly used for peptide immunoaffinity enrichment.

Directly to the digested peptide mixture, 15 μL of 1 M TRIS (pH 8, no. 15568-025) and 15 μL of 1x internal standard mix were added. Final IS concentrations for PIDD signature peptide internal standard are shown in Table S1. After protein extraction, 300 μL of extracted supernatant was removed and transferred to a new plate. Next, mAb-beads were directly added at the masses listed in Table S1. The resulting solution was incubated overnight at 4°C with agitation to allow for peptide capture by anti-peptide antibodies.

After incubation, mAb-beads were pulled down using a 96-well magnetic plate (Alpaqua Magnum EX, no. A000380) (Beverly, MA) and washed twice with 0.1 × PBS+ 0.01% CHAPS to remove off-target peptides. After each wash, mAb-beads were collected by the magnet before resuspension with additional wash buffer. After the final wash, they were collected by the magnet before addition of 30 μL of aqueous elution solution containing 5% AA + 3% ACN. Captured peptides were eluted from beads for 5 min at 1,000 rpm. Finally, beads were collected by the magnet and the eluted peptide was transferred to an LC/MS vial or a new 96-well plate (Abegene 96 well, no. AB-1058) (Chicago, IL) for analysis. Samples with less than the necessary DBS available were analyzed from either 4 or 5 × 3.125 mm punches. In these cases, all volumes were adjusted accordingly except for antibody input mass.

### Liquid Chromatography Mass Spectrometry

#### Seattle Children's Research Institute

LC-MS/MS of isolated peptide mixtures was performed on a Waters Xevo TQ-XS with Ionkey source and dual M-Class gradient and loading chromatography pumps (Milford, MA). Chromatographic solvents were A: H_2_O + 0.1% FA and B: ACN + 0.1% FA. As an initial step, peptides are loaded onto an M-Class Trap Symmetry C18 column (300 μM × 25 mm, 100Å, 5 μM) for 3 min with a constant flow of 98:2 A:B at 20 μL/min. After loading, the flow is reversed. Peptides are eluted from the trapping column and separated using a 150 μM x 100 mm BEH C18 ionkey (130Å, 1.7 μM). Gradient flow conditions are shown for both a 20 and a 2.5-min gradient (Table 1). A 300 μM × 50 mm BEH C18 ionkey (130Å, 1.7 μM) was used for the 2.5-min gradient. SRM transitions were acquired in unit resolution in both Q1 and Q3 quadrupoles. Dwell time was 5 ms with a 3 ms pause between mass ranges and the total cycle time was 1.5 s. Precursor and fragment masses for each peptide were chosen to generate the highest intensity transitions. Precursor mass, fragment mass, and collision energy were tuned to optimize the generated signal. Endogenous SRM traces for each peptide are shown in Figure S2.

**Table 1:** Liquid chromatography conditions for standard methods at SCRI and FHCRC and HT Immuno-SRM analysis at SCRI.

**SCRI standard**			**SCRI HT**			**FHCRC standard**		
**Time (min)**	**Flow (μL/min)**	**% B**	**Time (min)**	**Flow (μL/min)**	**% B**	**Time (min)**	**Flow (μL/min)**	**% B**
0	3	5	0	9	10	0	0.3	1
1	3	10	0.2	9	10	4	0.3	1
11	3	25	0.8	9	65	24	0.3	40
13	3	85	1	9	95	25	0.3	90
15	3	85	1.01	4	95	26	0.3	90
17	3	5	1.5	4	95	27	0.3	1
20	3	5	1.51	7	10	35	0.3	1
			1.7	7	10			
			2	9	10			
			2.5	9	10			

#### Fred Hutchinson Cancer Research Center

LC-MS/MS was performed using an Eksigent 425 LC and autosampler system with cHiPLC flex coupled to a SCIEX 5500 QTRAP mass spectrometer (Foster City, CA). Chromatographic solvents were A: H_2_O + 0.1% FA and B: 90% ACN + 0.1% FA in water. Peptides were loaded on a trap column (0.2 × 0.5 mm) with constant flow of 98:2 A:B for 3 min at 5 μL/min. The peptides were eluted and analyzed by nanoflow chromatography using a 15 × 0.075 mm column packed with Reprosil AQ C18 (3 μm) particles. Gradient parameters are shown in Table 1. SRM transitions were acquired unscheduled (i.e., without retention time scheduling) in unit/unit resolution using 5ms dwell times and 3ms pause. Optimized collision energies were obtained from Skyline software (49, 50). The gradient settings are shown in Table 1.

### Data Analysis

SRM data were analyzed using Skyline (MacCoss Lab, open source software, Seattle, WA, https://skyline.ms/project/home/begin.view). Specificity was assured by verifying equivalent retention times and relative transition intensities of endogenous and IS peptides. Concentrations of endogenous signature peptides were generated by comparing endogenous peptide signal to the signal of the isotopic IS added to the peptide extract at a known concentration. Statistical analyses were done using Graphpad Prism (San Diego, CA). Peptide reductions were analyzed using one-way ANOVA compared against the normal control for each peptide.

### Method Performance Assessment

A response curve was generated to establish assay linearity, as well as lower limits of detection (LLOD) and quantification (LLOQ). Punches from a normal control DBS (2 × 6.35 mm punches) were extracted and digested for each sample. After digestion, samples were pooled and re-aliquoted to generate a consistent endogenous peptide signal. To the aliquoted samples, eight different concentrations (0×, 0.05×, 0.1×, 0.5×, 1×, 2×, 5×, and 50×) of IS were added. Each concentration was added in triplicate. After IS addition, Immuno-SRM workflow was completed as described. After peptide elution, samples were split into two vials for LC-MS/MS analysis and run through two separate gradients (20 and 2.5 min) to compare the respective analytical values. Response curve samples were run through both the standard and HT gradients. LLOD was calculated using the following formula: LLOD = Mean_blank_ + 3 × SD_Low_, where Mean_blank_ is the mean signal from a triplicate blank injection and SD_Low_ is the standard deviation measured from an injection where the IS concentration is below the LLOQ. LLOQ was determined by the lowest point on the linearity curve with coefficients of variation (CV) < 20%.

Intra- and Inter-assay precision of the assay was determined by quantifying endogenous peptide concentrations across five separate days. Each day, five replicate assays were conducted through the entire Immuno-SRM process. CV was determined for within day (intra-day) and between day (inter-day) sample sets. CV samples were run through both the standard and HT gradients.

To study endogenous protein and peptide stability, the same DBS card was analyzed over time. Three separate DBS cards were stored at RT and 37°C and compared to a sample kept at −20°C to determine the effects of storage temperature. Peptide concentration measurements were made after 7 days of incubation. Each sample was analyzed in triplicate.

### Patient Sample Analysis

Patient samples were analyzed in a blinded fashion by generating mixed sample sets containing patients and controls. Full sample sets were run through the standard 20-min gradient for initial establishment of diagnosis. After analysis, patient condition was predicted by comparison to diagnostic cutoffs before unblinding. A subset of 17 samples and 20 normal controls were analyzed using the 2.5-min HT-gradient to examine the agreement of the standard and HT methods.

### Inter-laboratory Validation of Assay

Complete process validation, including punching, extraction, digestion, and Immuno-SRM analysis, was done at Fred Hutchinson Cancer Research Center (FHCRC). Assays were characterized for linearity and LLOQ by triplicate analysis of a response curve using eight levels of IS (100×, 10×, 2.5×, 2.5×, 2.5×, 2.5×, 2.5×, 2.5× dilution, respectively) spiked into the normal DBS background matrix. Lower limit of quantification (LLOQ) was determined by the lowest point on the curve with CV < 20%. Intra- and inter-assay repeatability were characterized by performing five complete process replicates of endogenous measurement of normal DBS controls over five separate days. Finally, a blinded sample set containing patients, normal controls, and negative controls was analyzed to compare measured concentrations and diagnoses between laboratories.

## Results

### Peptide Selection and Antibody Development

Signature peptide sequences chosen as representative biomarker peptides are listed in Table 2 along with molecular weights and parent and fragment ions used for quantitative analysis. Fragmentation patterns for WASP 274 and BTK 407 have been previously reported (26).

**Table 2:** Signature peptide information for primary and secondary markers including sequence, mass, and parent and fragment ions.

**Disease or cell target**	**Marker type**	**Protein**	**Peptide**	**Sequence**	**Mass (Da)**	**Parent ion (m/z)**	**Fragment ion (m/z)**
Wiskott-aldrich syndrome	Primary	WASP	WASP 274–288	AGISEAQLTDAETSK	1521.76	760.88 ++	[y10]−1063.5266+, [y9]−992.4895+, [y8]−864.4309+, [y7]−751.3468+, [y6]−650.2992+, [y3]−335.1925+, [b3]−242.1499+
X-Linked agammaglobulinemia	Primary	BTK	BTK 407–417	ELGTGQFGVVK	1135.63	567.81 ++	[y9]−892.4887+, [y7]−734.4196+, [y6]−677.3981+, [y5]−549.3395+, [y9]−446.7480++, [y8]−418.2373++
X-Linked chronic granulomatous disease	Primary	CYBB	CYBB 509–521	TLYGRPNWDNEFK	1639.767	547.2670 +++	[y8]−1049.4687+, [y4]−537.2667+, [y12]−769.8730++, [y11]−713.3309++, [y10]−631.7993++
Adenosine deaminase deficiency	Primary	ADA	ADA 93–101	EGVVYVEVR	1049.173	525.2849 ++	[y7]−863.4985+, [y6]−764.4301+, [y5]−665.3617+, [y3]−403.2300+, [b3]−286.1397+
DOCK8 deficiency	Primary	DOCK8	DOCK8 1272–1283	TSGIVLSSLPYK	1264.466	632.8610 ++	[y10]−1076.6350+, [y8]−906.5295+, [y7]−807.4611+, [y6]−694.3770+, [y5]−607.3450+, [b4]−359.1925+
Platelets	Secondary	CD42	CD42 128–137	LTSLPLGALR	1040.254	520.8268 ++	[y9]−927.5622+, [y8]−826.5145+, [y6]−626.3984+, [y4]−416.2616+
	Secondary	CD42	CD42 154–165	TLPPGLLTPTPK	1234.483	617.8739 ++	[y10]−1020.6088+, [y9]−923.5560+, [y8]−826.5033+, [y10]−510.8080++, [y9]−462.2817++
NK cells	Secondary	CD56	CD56 122–130	NAPTPQEFR	1059.128	530.2645 ++	[y7]−874.4417+, [y6]−777.3890+, [y5]−676.3413+, [y4]−579.2885+, [y7]−437.7245++, [y6]−389.1981++, [y5]−338.6743++

*Primary markers are used for direct diagnosis of the specific associated PIDD. Secondary markers provide information related to markers of hematopoiesis associated targets*.

Monoclonal antibodies against each sequence have been generated. Peptide sequences that had previously generated high quality pAbs (WAS 274 and BTK 407) for Immuno-SRM assays were used to launch mAb development for WAS and XLA. Final antibodies were chosen for their ability to capture endogenous signature peptides with high affinity.

### Method Performance Assessment

The multiplexed assay was characterized according to fit-for-purpose analytical validation criteria. Analytical figures including linearity, LLOD, LLOQ, intra-assay and inter-assay CV for standard gradient conditions, and stability are shown in Table 3. All peptides had LLOD and LLOQ values of <10 fmol except for CD42 128 which had an LLOD of 17.6 fmol and an LLOQ of 30 fmol. Linear range plots are shown in Figure S3. All peptide concentrations were reproducible with CVs < 20%. Typically, this is the accepted limit of variation between sample runs. Analytical figures for HT gradient analysis are shown in Table 4. The LLOD values were equivalent in between the 2.5- and 20-min runs while the LLOQ values increased in each case except for CD56 122, ADA 93, and CYBB 509 when moving to the HT method. Intra-assay CV tended to increase with a faster method, while remaining <20% for all peptides except for DOCK8 1272.

**Table 3:** Analytical figures of merit for standard PIDD gradient.

**Marker type**	**Protein**	**Peptide**	**LLOD (fmol)**	**LLOQ (fmol)**	**ULOD (fmol)**	**Intra-assay CV (Average, %)**	**Inter-assay CV (%)**	**Relative difference (RT, %)**	**Relative difference (37^**°**^C, %)**
Primary	WASP	WASP 274–288	5.6	9.4	937.5	9.7	9.7	11.6	−9.8
Primary	BTK	BTK 407–417	0.8	1.9	937.5	9.8	5.2	15.2	−18.9
Primary	CYBB	CYBB 509–521	4.2	7.5	7500.0	5.3	4.4	9.9	−19.0
Primary	ADA	ADA 93–101	0.8	3.8	3750.0	7.9	13.5	15.8	18.4
Primary	DOCK8	DOCK8 1272–1283	1.1	4.7	468.8	15.3	18.4	7.9	−9.6
Secondary	CD42	CD42 128–137	17.6	30.0	15000.0	5.4	10.9	25.5	27.9
Secondary	CD42	CD42 154–165	1.1	7.5	7500.0	6.1	5.8	14.0	−10.7
Secondary	CD56	CD56 122–130	2.7	3.8	1875.0	10.3	9.7	17.5	2.9

*Primary markers are used for direct diagnosis of the associated PIDDs. Secondary markers provide information related to markers of hematopoiesis associated targets*.

**Table 4:** Comparison of LLOD, LLOQ, and CV of the Standard 20-min and HT 2.5-min Gradients.

**Marker type**	**Peptide**	**LLOD (fmol)**		**LLOQ (fmol)**		**Intra-assay CV (Average, %)**		**Inter-assay CV (%)**	
		**20 min**	**2.5 min**	**20 min**	**2.5 min**	**20 min**	**2.5 min**	**20 min**	**2.5 min**
Primary	WASP 274–288	5.6	5.3	9.4	18.8	9.7	19.6	9.7	9.8
Primary	BTK 407–417	0.8	0.5	1.9	18.8	9.8	13.2	5.2	6.9
Primary	CYBB 509–521	4.2	0.6	7.5	7.5	5.3	7.6	4.4	3.2
Primary	ADA 93–101	0.8	6.9	3.8	3.8	7.9	11.6	13.5	13.2
Primary	DOCK8 1272–1283	1.1	1.6	4.7	18.8	15.3	29.5	18.4	11.0
Secondary	CD42 128–137	17.6	9.9	30.0	150.0	5.4	12.7	10.9	5.0
Secondary	CD42 154–165	1.1	2.0	7.5	15.0	6.1	7.2	5.8	4.5
Secondary	CD56 122–130	2.7	5.4	3.8	3.8	10.3	12.5	9.7	11.0

*Primary markers are used for direct diagnosis of the associated PIDDs. Secondary markers provide information related to markers of hematopoiesis associated targets*.

Additionally, there is a <20% change in peptide concentration over a 7-day period of storage at RT or 37°C relative to a sample stored at −20°C in all cases except for CD42 128. This platelet marker showed increases of 25.5 and 27.9% when stored at RT and 37°C, respectively.

### Normal Control Ranges

Immuno-SRM analysis of 175 normal control samples (125 for CYBB 509) was performed to set normal ranges. These ranges were used to establish diagnostic cutoff values by which patients could be identified. Average values, standard deviations (SD), and diagnostic cutoff values for each peptide in each condition are shown in Table 5.

**Table 5:** Average normal concentration values for signature peptides (*n* = 175, CYBB 509: *n* = 125) and current diagnostic cutoffs.

	**Average ± SD (pmol/L)**	**Diagnostic cutoff (pmol/L)**	**Diagnostic cutoff (SD)**
WASP 274	1629.6 ± 911.5	262.3	−1.50 SD
BTK 407	1164.9 ± 363.5	165.4	−2.75 SD
CYBB 509	2056.1 ± 830.6	187.4	−2.25 SD
ADA 93	2905.2 ± 1320.2	462.8	−1.85 SD
DOCK8 1272	365.3 ± 134.7	62.2	−2.25 SD
CD42 128	11545.4 ± 4067.2	3411.0	−2.00 SD
CD42 154	18523.0 ± 7534.3	7447.6	−1.47 SD
CD56 122	2493.9 ± 804.8	482.0	−2.50 SD

### Patient Samples

Signature peptide concentrations, Immuno-SRM and clinical diagnoses, genetic information and treatments are shown in Table 6. Five WAS DBS (patient samples 17-20-B) and five BTK DBS (patient samples 6-10) were carried over from a previous report for re-analysis with the extended signature peptide panel and under new analytical conditions to ensure fidelity of Immuno-SRM diagnosis (15).

**Table 6:** Patient information including measured concentrations of all signature peptides, diagnoses, genetic information, and present treatments.

**Pt**	**WASP 274 (pmol/L)**	**BTK 407 (pmol/L)**	**CYBB 509 (pmol/L)**	**ADA 93 (pmol/L)**	**DOCK8 1272 (pmol/L)**	**CD42 128 (pmol/L)**	**CD42 154 (pmol/L)**	**CD56 122 (pmol/L)**	**Immuno-SRM diagnosis**	**Clinical diagnosis**	**Gene**	**Mutation**	**Notes**
1	1478.1	2.7	3659.0	2039.7	432.1	16605.3	11732.6	4353.4	XLA	XLA	*BTK*	*BTK* c.1855C>G (p.P619A)	
2	1244.8	ND	2611.2	2992.8	430.4	17836.8	7.5	5413.0	XLA	XLA	*BTK*	N/A	
3	1906.5	631.5	4413.9	3586.6	533.5	25795.2	31025.1	4125.9	Normal^*^	XLA	*BTK*	*BTK* c.1630A>G (p.R544G)	^*^Diagnosed by BTK 545
4	1943.1	87.3	4554.5	4075.9	455.4	20969.1	12224.2	2284.1	XLA	XLA	*BTK*	*BTK* c. 1573C>G (p.R525G)	Brother of 5
5	1500.3	76.8	2959.7	4206.0	433.2	20434.3	10880.6	3618.6	XLA	XLA	*BTK*	*BTK* c. 1573C>G (p.R525G)	Brother of 4
6	1273.0	4.9	2306.6	1326.2	271.4	8547.4	26853.4	2620.2	XLA	XLA	*BTK*	*BTK* c.1889T>A (p.M630K)	
7	1162.2	5.7	1088.1	1581.2	185.7	7326.9	22085.1	1253.4	XLA	XLA	*BTK*	*BTK* c.1940T>C (p.L647P)	
8	1805.1	ND	3699.9	1358.8	436.6	8761.7	24798.0	2776.1	XLA	XLA	*BTK*	*BTK* c.1587_1589delA (p.N530Tfs26^*^)	Brother of 9
9	1728.9	ND	3769.7	1930.7	556.9	15184.3	29843.1	3830.4	XLA	XLA	*BTK*	*BTK* c.1587_1589delA (p.N530Tfs26^*^)	Brother of 8
10	1346.4	2.7	1221.0	1550.4	411.1	10008.0	28056.0	1759.2	XLA	XLA	*BTK*	*BTK* c.1940T>C (p.L647P)	
11	1511.4	0.4	N/A	1984.2	200.4	5411.2	7120.6	2117.6	XLA	XLA	*BTK*	*BTK* c.1567-13_1567-10delGTTT	
12	1035.2	82.6	2500.8	4346.4	336.9	10056.7	13776.0	3386.2	XLA	Normal	*BTK*	*BTK* c. 1573C>G (p.R525G) Carrier	Mother of 4 and 5
13	1834.8	455.1	N/A	2759.6	321.7	7613.2	9465.4	2057.0	Normal	Normal	*BTK*	*BTK* c.1567-13_1567-10delGTTT Carrier	Mother of 11
14	12.8	627.8	4483.9	7145.5	767.1	5979.4	3947.7	5481.1	WAS	WAS	*WAS*	*WAS* c.336_337insCC:F113Fs13^*^	
15	47.1	321.5	4995.4	2743.2	404.4	1389.3	2183.3	1665.3	WAS	WAS	*WAS*	*WAS* c.226_228delAAG	
16	30.6	258.3	3512.9	3590.1	360.7	1545.6	2607.1	2447.1	WAS	WAS	*WAS*	*WAS* p.R34X	
17	5.8	542.7	N/A	1725.6	75.5	2968.8	6607.8	1903.4	WAS	WAS	*WAS*	*WAS* c.756G>A (p.W252^*^)	
18	76.8	298.2	N/A	4459.5	117.3	1057.2	5614.3	2270.5	WAS	WAS	*WAS*	*WAS* c.223G>A (p.V75M)	
19	ND	591.5	N/A	3108.3	512.7	2667.0	7372.3	4333.0	WAS	WAS	*WAS*	*WAS* c.631C>T (p.R211^*^)	
20-A	86.4	627.0	N/A	3019.2	236.5	1378.3	2272.3	5674.2	WAS	WAS	*WAS*	*WAS* c.1453+2T>A	Pre-HSCT
20-B	1101.6	960.2	N/A	3307.8	240.1	6015.4	7593.9	8026.0	Normal	WAS	*WAS*	Normal HSCT donor	Post-HSCT
21	1271.1	907.8	30.2	5390.4	460.6	11137.4	18200.9	3973.5	X-CGD	X-CGD	*CYBB*	*CYBB* c.1010G>A (p.Trp337^*^)	
22	1202.1	1151.4	82.7	2636.4	360.7	13354.3	30910.7	4666.5	X-CGD	X-CGD	*CYBB*	N/A	
23	1325.9	1103.1	27.9	1592.1	310.5	13046.6	11325.0	4261.9	X-CGD	X-CGD	*CYBB*	N/A	
24	2406.0	1465.8	1172.6	2662.3	676.2	13438.6	29036.6	4359.1	Normal	AR-CGD	N/A	N/A	
25	866.4	714.0	1546.7	1501.1	18.2	6708.9	13978.7	2732.6	DOCK8 Def.	DOCK Def.	*DOCK8*	*DOCK8* c.54-4611_946del:c.3531-9_3531-8insCA	10% Revertant PBMC
26	790.3	1417.1	1417.4	6540.7	408.2	20157.3	32062.6	3380.6	Normal	ADA Def.	*ADA*	*ADA* c.96-2A>AG + c.755T>TA (p.L252LQ)	Transfusion
27	2913.0	826.5	14972.6	3232.1	563.5	13920.0	8345.1	3128.6	Normal	ADA Def.	N/A	N/A	Transfusion
28	905.9	719.3	1129.3	15.6	215.8	8421.4	18474.0	3661.6	ADA Def.	ADA Def.	*ADA*	N/A	PEG-ADA ERT

**Figure 1:**
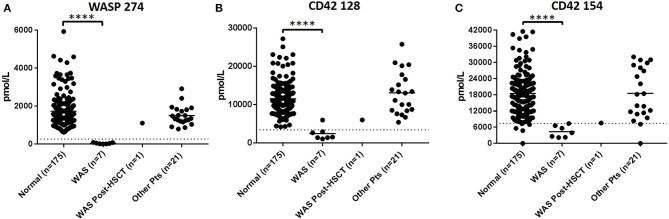
Immuno-SRM analysis of WASP 274 in WAS patients **(A)**. Platelet markers CD42 128 **(B)** and CD42 154 **(C)** show corresponding changes in platelet levels. ****p < 0.001.

In DBS of WAS patients (*n* = 8), signature peptides were significantly reduced relative to healthy controls (*p* < 0.001). Normal concentrations of WASP 274 were 1629.6 ± 911.5 pmol/L in control samples. All untreated patients were below the WASP diagnostic cutoff. These patients also tended to exhibit a significant reduction in the concentrations of platelet markers CD42 128 and 154 (Figure 1). For CD42 128, six of seven untreated patients were below diagnostic cutoffs. For CD42 154, all untreated patients were below the defined diagnostic cutoff. CD42 154 however, had a greater number of false positives and samples with no detectable peptide. This is potentially due to unknown polymorphisms causing mass changes and interfering with peptide detection. Finally, samples 20-A and 20-B were collected from the same patient before and after curative HSCT. After HSCT, this patient had WASP 274, CD42 128, and CD42 154 levels above the diagnostic cutoff for each peptide (Table 6). Finally, patient 14 had CD42 128 levels above the diagnostic cutoff. All non-target peptides were within the normal range for these patients and no other patient had levels of WASP 274 or CD42 128 below diagnostic cutoffs.

**Figure 2:**
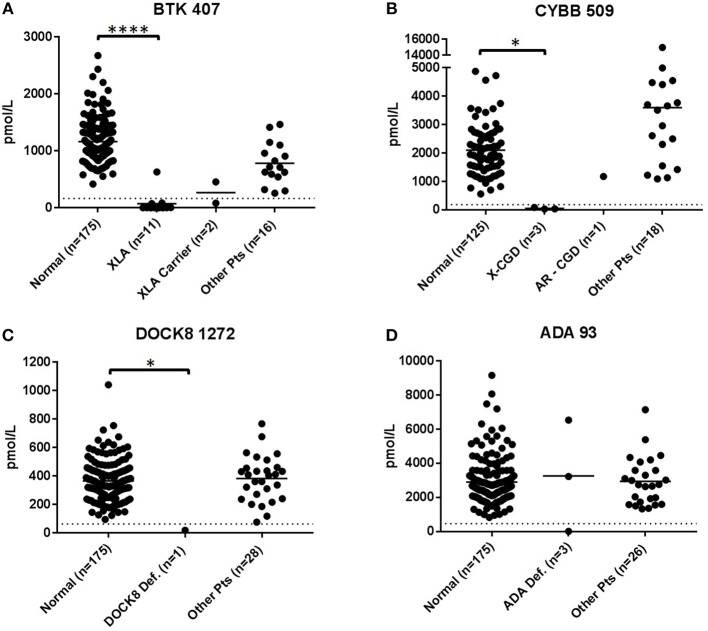
Immuno-SRM analysis of primary signature peptides BTK 407 **(A)**, CYBB 509 **(B)**, DOCK8 1272 **(C)**, ADA 93 **(D)** in XLA, XL-CGD, DOCK8 deficiency, and ADA deficiency patients. *p < 0.05, ****p < 0.001.

XLA patients DBS (*n* = 11) except for patient 3 had levels of BTK 407 significantly reduced from normal controls (*p* < 0.001) and below diagnostic cutoffs. BTK peptide concentrations relative to normal controls are shown in Figure 2A. Patient 3 was deficient in a second XLA signature peptide BTK 545 (Figure S4). All non-target peptides were within the normal range for these patients and no other patient had BTK 407 below diagnostic cutoffs. Of the carriers with BTK mutations (*n* = 2), one sample was above diagnostic cutoff and one was below.

DBS of confirmed XL-CGD patients (*n* = 3) were found to have CYBB concentrations reduced from normal. The average concentration of CYBB in XL-CGD patients was 46.9 pmol/L (Figure 2B). AR-CGD patient 24 had a CYBB concentration of 1172.6 pmol/L well above the diagnostic cutoff of 187.4 pmol/L CYBB 509. All non-target peptides were within the normal range for these patients.

The DOCK8 deficiency patient (DBS 25, *n* = 1) (Figure 2C) had a DOCK8 1272 peptide concentration of 18.2 pmol/L. This is significantly reduced when compared to a normal control average of 365.3 pmol/L (*p* < 0.05) and a diagnostic cutoff of 62.2 pmol/L. All non-target peptides were within the normal range for this patient and no other patients had below cutoff levels of DOCK8 1272.

DBS from ADA deficiency patients 26–28 (*n* = 3) had variable ADA 93 values (Figure 2D). Patient 28 had ADA 93 concentration of 15.6 pmol/L, well below the diagnostic cutoff set at 462.8 pmol/L. Patients 26 and 27 had peptide concentrations above cutoff levels. Patient 27 had a near average ADA 93 concentration of 3232.1 pmo/L while patient 26 had an elevated peptide concentration at 6540.7 pmol/L. All three patients were undergoing some form of treatment, with Patient 28 currently on PEG-ADA ERT and patients 26 and 27 undergoing regular RBC transfusions to manage their ADA deficiency. No other PIDD patient or normal control samples had ADA 93 levels below cutoff concentrations.

**Figure 3:**
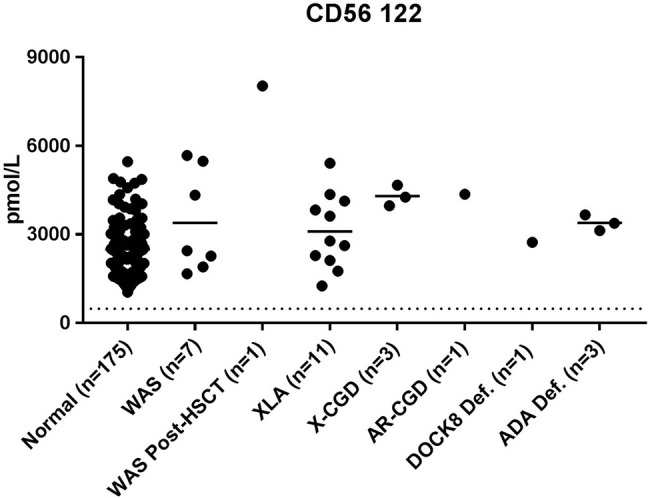
Levels of NK Cell marker CD56 122 found in DBS of PIDD patients.

All patient samples had NK cell marker CD56 122 levels above the diagnostic cutoff determined by normal controls (Figure 3).

### Cross-Validation of Immuno-SRM for PIDD in a Second Laboratory

Analytical validation conducted in a separate laboratory was carried out at Fred Hutchinson Cancer Research Center (FHCRC). This included analysis of the linear range of the assay, limits of detection and between-run and between-day variation (Table 7). Analysis at FHCRC found LLOQ values below 3.84 fmol in all cases, with five of the peptides measured having LLOQs below 1 fmol. This is 4-15.6x lower than those measured at SCRI excluding ADA 93 where the LLOQ was found to be ~39x lower. Coefficients of variation for the assay were all <20% except for the inter-assay CV for ADA 93 which was significantly more variable at 31.5%.

**Table 7:** Immuno-SRM analytical validation at FHCRC including lower limits of quantification (LLOQ), Upper limit of detection (ULOD), and Intra- and Inter-assay Co-efficients of Variation (CV).

**Protein**	**Peptide**	**LLOQ (fmol)**	**ULOD (fmol)**	**Intra-assay CV (Average, %)**	**Inter-assay CV (%)**
WASP	WASP 274–288	0.24	375	9.0	6.8
BTK	BTK 407–417	0.24	375	11.0	7.1
ADA	ADA 93–101	0.96	1,500	12.8	31.5
DOCK8	DOCK8 1272–1283	0.3	187.5	11.8	10.3
CD42	CD42 128–137	3.84	6,000	11.5	5.8
CD42	CD42 154–165	1.92	3,000	16.5	13.4
CD56	CD56 122–130	0.48	750	13.1	7.2

**Figure 4:**
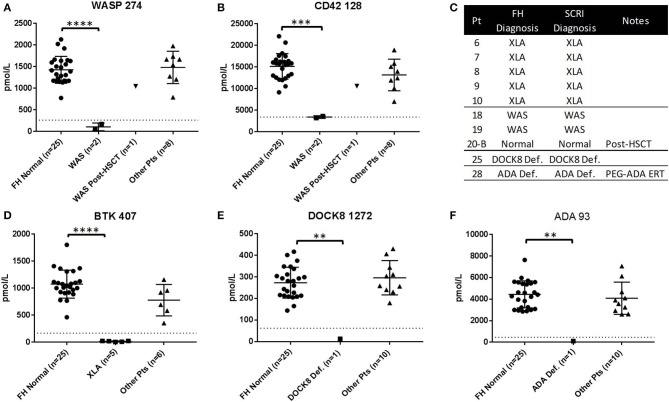
Results of blinded sample Immuno-SRM analysis conducted by alternate laboratory. Complete process replication included DBS card punching, extraction, digestion, enrichment and MS analysis. Normal Controls (*n* = 25) and patients (*n* = 11) are compared against the diagnostic cutoff established by SCRI. Signature peptide values for WASP 274 **(A)** and CD42 128 **(B)**, BTK 407 **(D)**, DOCK8 1272 **(E)**, and ADA 93 **(F)** are shown in normals and PIDD patients. Comparative diagnosis shows agreement in patient identification **(C)**. FH, Fred Hutchinson Cancer Research Institute; SCRI, Seattle Children's Research Institute. **p < 0.01, ***p < 0.005, ****p < 0.001.

To study the ability of Immuno-SRM diagnostic results to be produced in multiple locations, a blinded sample set of normal controls (*n* = 25) and patient samples (XLA: *n* = 5, WAS: *n* = 3, XL-CGD: *n* = 1, DOCK8: *n* = 1, ADA: *n* = 1) were analyzed by a separate laboratory at FHCRC (Figure 4). In every patient case, the primary diagnostic markers (Figures 4A,D–F) were reduced below the diagnostic cutoffs established by SCRI. In WAS patients (*n* = 3), untreated patients had platelet marker CD42 128 levels significantly reduced from control and below or near SCRI cutoffs and increased to normal levels after HSCT (Figure 4B, patient 20-B). Therefore, patient diagnoses agreed between the two laboratories (Figure 4C). One XL-CGD patient analyzed has normal levels of all peptides as CYBB 509 was not measured (Table S2). Additional markers are shown in Figure S5 and patient data is shown in Table S2.

### High-Throughput (HT) Analysis of Patient Samples

**Figure 5:**
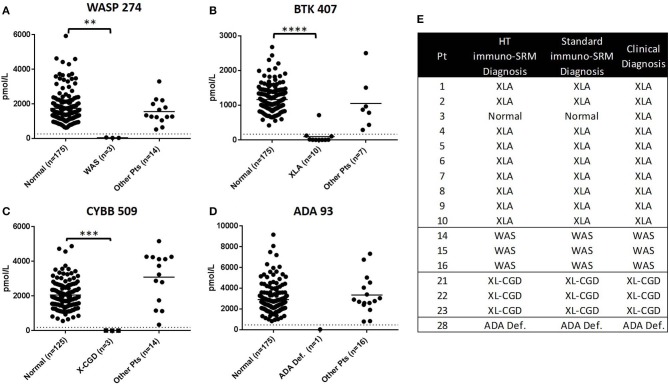
Comparison of PIDD patient identification using HT and standard Immuno-SRM. Signature peptide concentrations measured by HT-Immuno-SRM are shown in patients for WASP 274 **(A)**, BTK 407 **(B)**, CYBB 509 **(C)**, and ADA 93 **(D)**. Comparison of patient identifications are shown in **(E)**. **p < 0.01, ***p < 0.005, ****p < 0.001.

To demonstrate the potential for the method to be adapted to high-throughput screening, a subset of 17 patient samples (XLA: *n* = 10, WAS: *n* = 3, XL-CGD: *n* = 3, ADA Deficiency, *n* = 1), were analyzed using an optimized 2.5-min HT gradient amenable to population screening. No additional false positives were created with a HT analysis and diagnoses matched that of the standard LC-MS/MS method (Figure 5). High CVs were the reason for excluding DOCK8 1272 from HT method analysis.

## Discussion

We have demonstrated that Immuno-SRM is a highly sensitive multiplexed assay that correctly identifies patients with five genetically defined PIDDs by directly quantifying low abundance proteins present in DBS. Patients with ADA deficiency, DOCK8 deficiency, WAS, XLA, and XL-CGD can be screened simultaneously. New and supportive information is gained through analysis of secondary (i.e., not directly diagnostic) protein markers CD42 for platelets and CD56 for NK cells. The data also indicate that this method is highly sensitive, is reproducible over time, has a wide linear range, and is easily transferrable.

We previously reported using pAbs for the screening of WAS, XLA, and SCID (15). Since pAbs are a collection of antibodies that recognize different antigenic epitopes, only a subset of antibodies present may bind the peptides of interest. For this reason, there may be antibody present that does not recognize target peptide antigens causing a need for increased antibody mass to achieve acceptable results. Furthermore, the pAbs could cause increased off-target binding and result in increased complexity of the enriched peptide mixture. In addition, pAbs cannot be consistently reproduced because of their heterogeneity. The generation of mAbs makes possible the selection of an antibody that consistently recognizes a single epitope and straightforward assay optimization. Additionally, mAbs can be sequenced and reproduced to allow for the creation of a consistent, renewable assay reagent that can be subjected to rigorous quality control and achieve identical results overtime. Importantly in the cases of WAS and XLA, mAb reagents continued to produce analytical performance, limits of quantification, limits of detection, and variability, that are acceptable for clinical assays. The mAbs generated are expected to achieve stable results over time in a diagnostic laboratory and can be produced at scale for population screening.

To ensure the maintenance of mAb diagnostic performance in the new assays and analytical workflows, 5 WAS DBS (patient samples 17-20-B) and 5 XLA DBS (patient samples 6-10) were subjected to a new analysis with the expanded Immuno-SRM PIDD peptide panel. This analysis showed that WAS and XLA diagnoses were consistent and unaffected by the transition from *pAb* to *mAb* reagents. Additionally, an increased number of primary signature peptides in the immuno-SRM analysis leads to a concordant increase in the potential for false positives. Even with the inclusion of 3 additional primary markers for XL-CGD, DOCK8 and ADA deficiency, the Immuno-SRM assay remained entirely specific with only the affected peptide being reduced below diagnostic levels. Finally, additional disease-state information was gained about these samples from CD42 platelet markers and CD56 NK cell markers, as discussed below. These statements remained true in both the standard and HT-Immuno-SRM analytical methods presented here.

Multiplexed Immuno-SRM screening was able to successfully identify every confirmed case of WAS (*n* = 8) (Figure 1). Primary signature peptide WAS 274 was significantly reduced when compared to healthy normal controls and below the diagnostic cutoff set for the assay. As a consequence of their disease, WAS patients almost ubiquitously exhibit low numbers of platelets (44). As expected, secondary platelet markers CD42 128 and CD42 154 were also reduced in WAS patients, consistent with a reduction in circulating platelets. Patient sample 14 was found to be the only WAS patient with levels of CD42 128 that, while low, were above the diagnostic cutoff. It is not known if patient 14 had undergone splenectomy or platelet transfusion at the time of analysis. In this case, levels of CD42 154 reduced into the suspected patient range providing an example of the complementarity of these markers. CD42 signature peptides are therefore representative of platelet levels in DBS and can be used as a supportive marker for WAS. These two pieces of data taken together strengthen the cases for a diagnosis of WAS and show that multiplexing secondary immune system markers can provide an informative view of disease processes beyond direct PIDD diagnosis. Interestingly, when the same patient was analyzed before (patient 20-A) and after curative HSCT (patient 20-B), both primary WASP 274 and secondary platelet markers CD42 128 and CD42 154 were elevated above the diagnostic cutoff following HSCT.

Applying BTK 407 as a single primary marker led to the identification of all but one molecularly confirmed case of XLA (*n* = 11) (Figure 2). XLA patients were represented a set of 7 different disease-causing mutations. Concentrations of BTK 407 were below their respective diagnostic cutoffs in all cases except for patient 3. In this case, mAb enrichment of BTK 407 showed concentrations in the established normal range. The disease-causing mutation in this case (p.R544G) has been shown, in another reported patient, to reduce enzyme activity but not protein concentrations (51). This mutation is near a second mutation, p.Y551N, that has been previously found by Immuno-SRM not to affect BTK concentration when using signature peptide BTK 407 (15). However, a second BTK signature peptide, BTK 545, which included the site of the point mutation readily allowed for patient identification in both cases (Figure S4). In the case of patient 3, BTK 545 levels are not detectable. Signature peptides which contain sites of mutations are not detectable by MS due to the mass shift associated with an amino acid change. This causes a change in the total peptide mass which renders the mutated peptide undetectable in the mass spectrometer and leads to an absent wild-type biomarker signal. The amino acid change in patient 3, p.R544G eliminates the tryptic cleavage site directly before the BTK 545 biomarker peptide. This change blocks BTK 545 peptide release by trypsin digestion and similarly causes an absence of the wild type endogenous BTK 545 due to the molecular weight difference of the additional amino acids. This shows that using multiple signature peptides can increase the likelihood of patient identification, particularly if there are known mutations which would cause false negative by Immuno-SRM.

The above cases demonstrate one way in which misdiagnosis may occur by Immuno-SRM. Mutations which affect protein activity but not protein concentration have the potential to appear as false negatives after analysis. Continued analysis of patient DBS samples from a broad diversity of genetic background are underway to establish correlations between genetic mutation and measured Immuno-SRM protein concentration. This kind of survey will allow for generation of additional signature peptides. Alternatively, it is possible that specific lymphopenias will reduce the levels of proteins that are present predominantly in those cell types, with the potential to cause false positive results or result in a misdiagnosis. We have not yet seen a case of this but are continuing to develop cell-specific secondary markers to reveal lymphopenias where they exist.

An investigation of the BTK carriers (*n* = 2) included in the analysis suggests that the protein concentration is dependent on the types of mutation present in the index case (Figure 2). BTK analysis of carrier DBS 12 showed a BTK 407 level below diagnostic cutoffs, similarly to her two sons' DBS 4 and 5, possibly as a result of X-chromosome inactivation. Conversely, carrier patient 13, had BTK 407 concentrations of 455.1 pmol/L, well above the diagnostic cutoff of 165.4 pmol/L. It is therefore likely that the ability of Immuno-SRM to identify carriers will be dependent not only on the mutation's effect on protein stability, but also on the random X-chromosome inactivation pattern present in platelets and in nucleated blood cells other than B-cells of carrier females.

X-linked CGD patients (*n* = 3) were also readily identified using Immuno-SRM analysis of a CYBB signature peptide (Figure 2). CYBB 509 concentrations for patients 21-23 were well below the diagnostic cutoff of 187.4 pmol/L. This analysis will be specific to XL-CGD patients with mutations in the *CYBB* gene but not for autosomal recessive (AR) forms of the disease, which are brought on by loss of other components in the NADPH complex. This is evident in the analysis of AR-CGD patient 24 who showed normal levels of CYBB 509 (Table 6). Other signature peptides representing CYBA or NCF1-3 could be added for individual NADPH components to identify AR forms of CGD. Because NADPH oxidase measurement by flow cytometry requires venous blood drawn by venipuncture and neutrophils have a limited viability, Immuno-SRM from DBS represents a powerful alternative technology for clinical screening.

Analysis of primary markers for DOCK8 deficiency show that Immuno-SRM can readily differentiate a patient from controls (Figure 2). For analysis of DOCK8 1272, healthy controls showed an average peptide concentration of 365.3 ± 134.7 pmol/L. A diagnostic cutoff of was set at 62.2 pmol/L or 2.25 SD below average concentrations. The DOCK8 sample analyzed had a very low concentration of DOCK8 1272 by these methods when measuring from DBS. Interestingly, the patient studied here was found to have revertant phenotype with ~10% DOCK8 positive PBMCs by flow cytometry. Diagnosis of DOCK8 deficiency can often be difficult due to reversion, which needs specialized flow cytometry workflows so a robust assay capable of clearly differentiating most or all patient phenotypes, regardless of reversion, would greatly simplify identification (52). In addition, sequencing can be challenging due to the frequent presence of large deletions in one allele. It is possible that Immuno-SRM diagnostics will be more tolerant of reversion because this assay is a global analysis of all cells present in the DBS. Thus, while certain PBMC subsets may exhibit revertant expression of DOCK8, overall the total protein concentration is dramatically reduced. Because previous studies have suggested that nearly all DOCK8 patients have very low DOCK8 protein expression (41), we expect that most patients will be screened positive by Immuno-SRM. All other patients showed normal levels of DOCK8 1272 suggesting that this biomarker is characteristic for DOCK8 Deficiency.

Each ADA deficiency patient (*n* = 3) screened was on some form of enzyme replacement treatment (ERT) for their disorder and data suggests the form of the treatment impacted the ability of Immuno-SRM to identify patients (Figure 2). Patient 28 was correctly diagnosed using signature peptide ADA 93 despite being on pegylated-ADA ERT (Adagen). In this patient, ADA levels were extremely low at 15.6 pmol/L and well-below cutoff values of 462.8 pmol/L. The recombinant ADA enzyme used for treatment is bovine in origin and does not interfere with a quantification of endogenous human ADA by Immuno-SRM (53). Patients 26 and 27 from Brazil, however, were being treated with erythrocyte transfusion to provide exogeneous enzyme since RBCs have significant levels of ADA (54). With a half-life of 90 days, ADA-deficient patients who receive RBC transfusions at weekly intervals will have stable levels of RBC-associated human ADA in their blood and as a consequence appear normal in the Immuno-SRM assay. This is consistent with the ADA 93 levels measured by Immuno-SRM of 6540.7 and 3232.1 pmol/L in patients 26 and 27, respectively. The protein levels in patient 26 are in fact significantly elevated from the average normal values of 2905.2 ± 1320.2 pmol/L ADA 93 which may be due to an increased RBC number upon transfusion. While we do not have access to a further immunologic or clinical workup on these patients, the results suggest that Immuno-SRM may be sensitive to specific treatments and their effects. The assay also has the potential to function as a tool for determining the efficacy of treatments such as gene therapy or HSCT or measuring pharmacokinetic profiles of exogenously delivered protein therapeutics.

Additional evidence of ADA treatment here may come in the form of normal NK cell marker CD56 122 concentrations (Figure 3). ADA deficiency is a T-B-NK- type of SCID that would normally result in a reduction of NK cell concentrations in patients. Patient 28 had been receiving PEG-ADA (Adagen) treatment at the time of sample collection which has been shown to increase lymphocyte concentrations in patients with treatment (53). Likewise, patients 26 and 27 transfusion treatments can increase absolute lymphocyte numbers (55). Together, these data provide potential information about the success of therapeutic intervention and the potential consequences on the hematopoietic and immune system.

The concentration values reported (pmol/L) are based on detection of the tryptic signature peptides and standards. Absolute quantifications of the signature peptides are dependent on complete recovery of the tryptic peptides following enzymatic digestion. While further work is necessary to characterize the extent of peptide recovery following digestion, the work described herein shows enzymatic digestion of the targeted proteins is robust, reproducible, and consistent.

For successful implementation as a diagnostic or population screening tool, Immuno-SRM analysis must be instituted and replicated by multiple laboratories. To test the replicability of Immuno-SRM analysis of PIDD patients, the full process of the assay was reproduced at FHCRC. The entire Immuno-SRM assay was conducted including extraction, digestion, signature peptide enrichment, and LC-MS/MS analysis with separate laboratory personnel and on a different instrument. This included analytical validation, including LOQ, intra-assay CV, and inter-assay CV, as well as a blinded analysis of normal controls and patient samples. CV measurements were all found to be <20% except for the inter-assay CV for ADA 93 (Table 7). This shows the analytical robustness of Immuno-SRM workflows and the high reproducibility of the assay. LLOQs measured at FHCRC were 3.9-15.6x lower than at SCRI in all cases expect for ADA 93 where the quantification limit was reduced by a factor of 39. This difference is potentially due to the lower flow rate and longer runtime of the FHCRC assay resulting in higher detection sensitivity. Measured peptide concentrations were well correlated between the two institutions with correlation plot *R*^2^ values of 0.72-0.93 for primary diagnostic markers and 0.52–0.96 for secondary markers (Figure S6). Better correlation for direct diagnostic primary markers suggests that Immuno-SRM is highly reproducible as a clinical diagnostic from DBS.

Analysis of a sample set including normal control and patient DBS (XLA: *n* = 5, WAS: *n* = 3, XL-CGD: *n* = 1, DOCK8: *n* = 1, ADA: *n* = 1) found a complete diagnostic agreement between institutions (Figure 4). Peptide concentrations measured at FHCRC for XLA, WAS, DOCK8, and ADA deficiency primary markers were found to be reduced below diagnostic cutoffs established at SCRI and no false positives were created. One XL-CGD patient was included in the blinded analysis and found to have normal levels of all primary diagnostic markers as CYBB 509 was not measured at FHCRC. Platelet marker CD42 128 levels were significantly reduced in WAS patients without HSCT and normalized in the post-HSCT patient 20-B. This complete reproduction of Immuno-SRM assay results with two complete workflows at two separate institutions shows the significant potential of translating Immuno-SRM into clinical and population analysis.

While a 20-min runtime will be sufficient for Immuno-SRM analysis to be used as a clinical diagnostic tool, this analysis time will need to be significantly reduced in order to achieve the sample throughput necessary for NBS. To address this problem, we have designed an HT gradient that utilizes a 2.5-min sample runtime. Peptide transitions and retention time windows were optimized to maintain sufficient peptide signal and separation within a short analytical run. Overall, because of MS scan rates, it was necessary to reduce the number of monitored transitions, therefore reducing overall signal. The transitions used for this analysis are in Table S3. Despite this reduction, the limits of detection HT methods were similar in all cases except for ADA 93, CD42 154 and CD56 122 in which they were significantly higher (Table 4). In contrast, LLOQs for all peptides except for CYBB 509, ADA 93, and CD56 122 increased several-fold. These results are likely due to a reduced overall signal with HT analysis because of decreased transitions monitored and increased peptide co-elution and may be solved by using increased DBS input in an HT setting. Intra-assay CV increased in most cases but remained below 20% variation in every case except for DOCK8. DOCK8 1272 concentration analysis was poorly reproduced in HT analysis due to shifting peptide retention times and loss of resolution. Increases in peptide LLOQ and assay CV is possibly due to the reduction in monitored transitions or reduction in the number points measured across each peak with a faster run. Under the current gradient conditions, DOCK8 1272 would be a poor signature peptide candidate for NBS and needs to be analyzed under standard conditions. Additional candidate peptides for DOCK8 will be investigated for HT analysis.

A blinded set of samples from previous runs were analyzed by 2.5-min HT-gradient methods (Figure 5). Of the 17 patients analyzed in this sample set (XLA: *n* = 10, WAS: *n* = 3, XL-CGD: *n* = 3, ADA, *n* = 1), the indicated conditions matched that of the standard gradient. Results for these patients and normal controls are shown in Figure 5. For the primary markers WASP 274, BTK 407, CYBB 509, and ADA 93, no false positives were created by HT analysis. These results suggest that Immuno-SRM and the currently studied peptide biomarkers are amenable to an HT analysis required for population-based NBS studies.

These target PID disorders were chosen as representing strong potential candidates for newborn screening. They are relatively frequent disorders for which effective treatments, including prophylactic antibiotics and IV immunoglobulin, or curative options, including HSCT or gene therapy, exist. Without a robust newborn screening method, it often takes a prolonged time before these relatively rare congenital PIDDs are suspected and steps taken to establish the correct diagnosis and initiate effective treatments or curative procedures. During this time, patients are prone to life-threatening infections and other negative consequences of their untreated conditions within the first year of life. Genetic counseling for families identified by NBS can also be of significant value. These facts suggest that the PIDDs included in this study are excellent candidates for newborn screening and would bring significant benefit to the patient population and healthcare system. Immuno-SRM is an attractive potential platform for NBS screening of PIDDs because it is operationally simple, rapid, low cost, and multiplexed to include multiple conditions per patient in a single run from DBS. A 2.5-min HT run equates to a runtime of 2 conditions per minute or 3.2 biomarkers per minute if secondary cell markers are included to provide clinicians with increased context upon referral.

Here we report that Immuno-SRM has the potential to effectively identify 5 congenital PIDDs simultaneously from DBS, while providing additional context about the immune system. While the biomarker peptides themselves can be robustly measured across all samples and in methods relevant to both clinical diagnosis and NBS, increased numbers of patient DBS for each disorder are needed to define the diagnostic ability of the assay across a range of mutation and carrier status. Additionally, a large scale pilot study is being planned to more accurately predict the sensitivity and specificity of the assay. Straightforward testing for a number of relatively rare immune disorders would create significant diagnostic value when immunodeficiencies are suspected. This PIDD Immuno-SRM PIDD panel is able to produce clear results in the patients studied for multiple conditions in a highly-multiplexed overnight assay and can be further extended to other PIDDs where the gene product is frequently absent (e.g., X-linked Lymphoproliferative Disease 1 and 2, AR-CGD, Hyper IgM Syndrome, Ataxia Telangiectasia, etc.). Based on previous work, significantly higher multiplex levels are possible (37). Immuno-SRM extraction, digestion, and enrichment are operationally straightforward and, in most cases, amenable to current clinical workflows. The ability to perform this assay from small blood volumes extracted from DBS is important and convenient as DBS cards can be collected non-invasively and mailed easily at room temperature. An Immuno-SRM result suggestive of PIDDs would be invaluable in determining the need for further testing and genetic sequencing. In addition, the assay can provide contextual secondary information about the immune system analogous to flow cytometry or lymphocyte subset information. Immuno-SRM provides clear results for diseases that can often be difficult or time-consuming to diagnose and is a robust addition to current clinical workflows.

## Data Availability Statement

The raw data supporting the conclusions of this article will be made available by the authors, without undue reservation, to any qualified researcher.

## Ethics Statement

The studies involving human participants were reviewed and approved by the Institutional Review Board of Seattle Children's Hospital, the Fred Hutchinson Cancer Research Center, NIAID/NIH and the Universidade Federal de Uberlândia. Written informed consent to participate in this study was provided by the participants' legal guardian/next of kin.

## Author Contributions

CC was involved in acquisition of clinical patient data, drafting the manuscript, revising the manuscript, creating and editing the table and figures, and preparing the manuscript for publication. FY, RD, and JW were involved in peptide analysis performance, and drafting multiple sections of the manuscript. HO, AF, HS, TT, and GS provided the samples and were involved in revising the manuscript. SH as a senior author designed the study and was involved in data analysis, interpretation, and revising the manuscript. AP was involved in experimental design, data interpretation, and manuscript revision. All authors revised the manuscript critically for important intellectual content and gave final approval of the version to be published.

### Conflict of Interest

SH has received compensation and sponsored travel from Alexion Pharmaceutical Company. The remaining authors declare that the research was conducted in the absence of any commercial or financial relationships that could be construed as a potential conflict of interest.

## Supplementary Material

Figure S1 Fragment Spectra for each target peptide with selected transitions.

Figure S2 Endogenous MRM traces for signature peptides.

Figure S3 Linearity curves for each signature peptide. High fmol range plots **(A)** represent the full analyzed concentration range. Low fmol range plots **(B)** represent enlarged low concentration regions from A plots.

Figure S4 Analysis of XLA patients with secondary signature peptide BTK 545. ND, Not Detectable. Diagnostic cutoff was set at 1.75 SD below the normal mean.

Figure S5 Normal Control and patient DBS peptide concentrations generated at FHCRC for secondary markers CD56 122 and CD42 154.

Figure S6 Correlation plots for measured peptide concentrations between Fred Hutchinson Cancer Research Institute (FHCRC) and Seattle Children's Research Institute.

Table S1 Stock and final internal peptide concentrations and monoclonal antibody bead mass per sample for each target peptide.

Table S2 Peptide concentration data for patients measured at FHCRC.

Table S3 High-throughput gradient transitions for signature peptides. Primary markers are used for direct diagnosis of the specific associated PIDD. Secondary markers provide information related to markers of hematopoiesis associated targets.
