# Supplementary figures and images for: Multiplexed Proteomic Analysis for Diagnosis and Screening of Five Primary Immunodeficiency Disorders From Dried Blood Spots
Source: Front Immunol. 2020 Apr 1;11:464. doi: 10.3389/fimmu.2020.00464 (PMC7141245; doi:10.3389/fimmu.2020.00464)

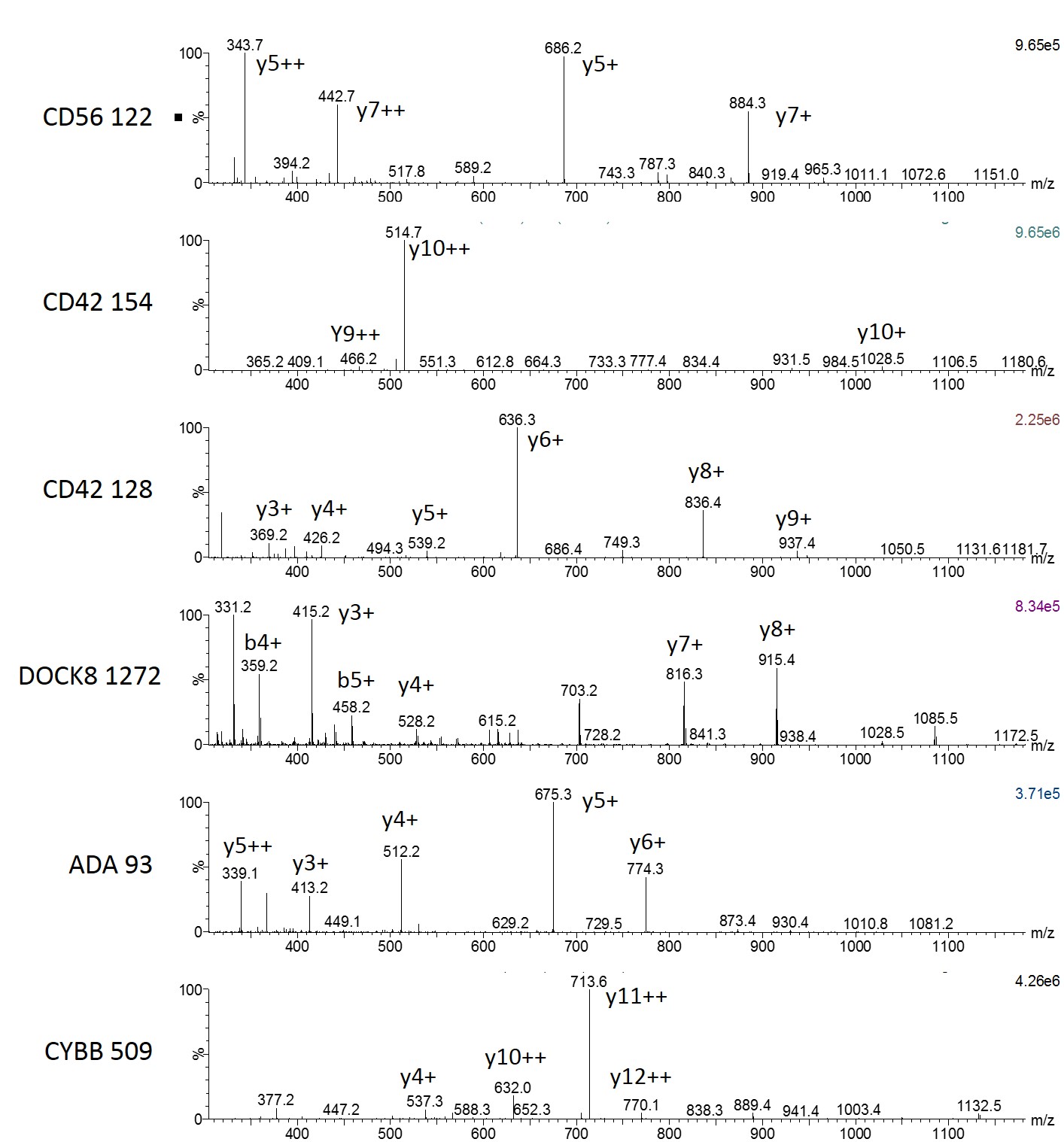

Supplement: Figure S1 — Fragment Spectra for each target peptide with selected transitions. [file Image_1.jpg]

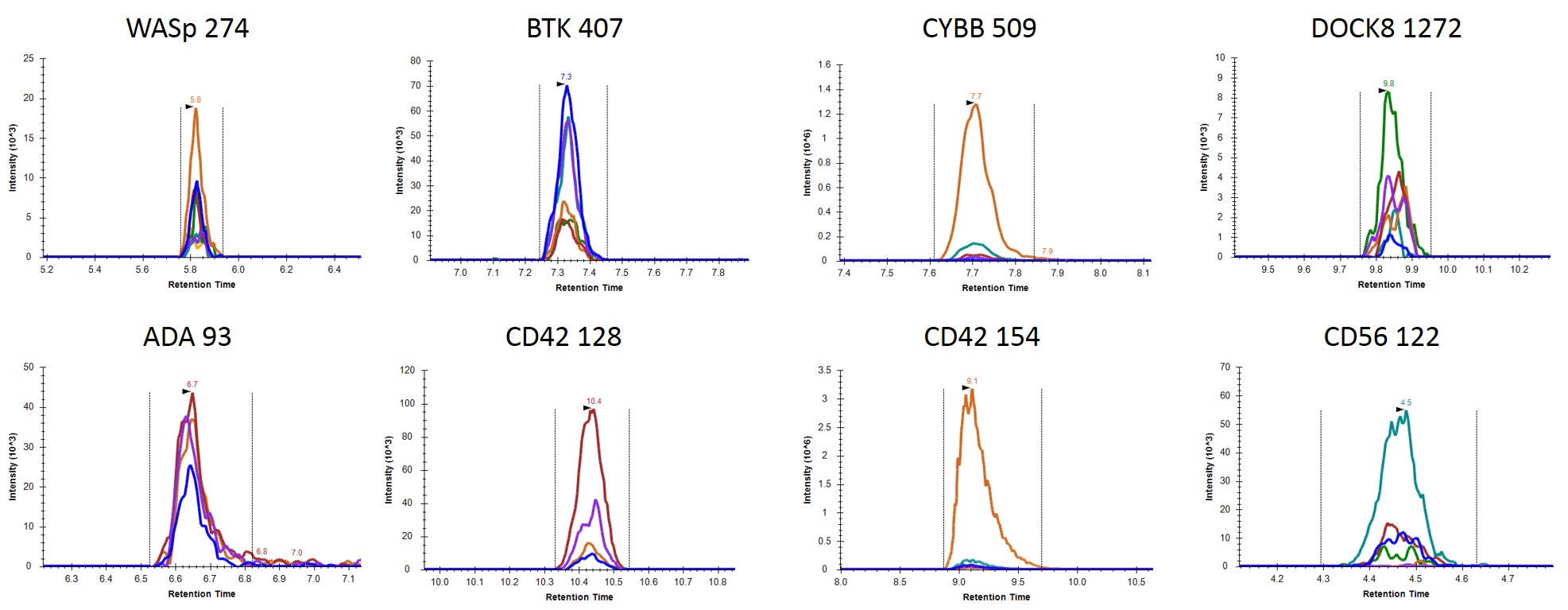

Supplement: Figure S2 — Endogenous MRM traces for signature peptides. [file Image_2.JPEG]

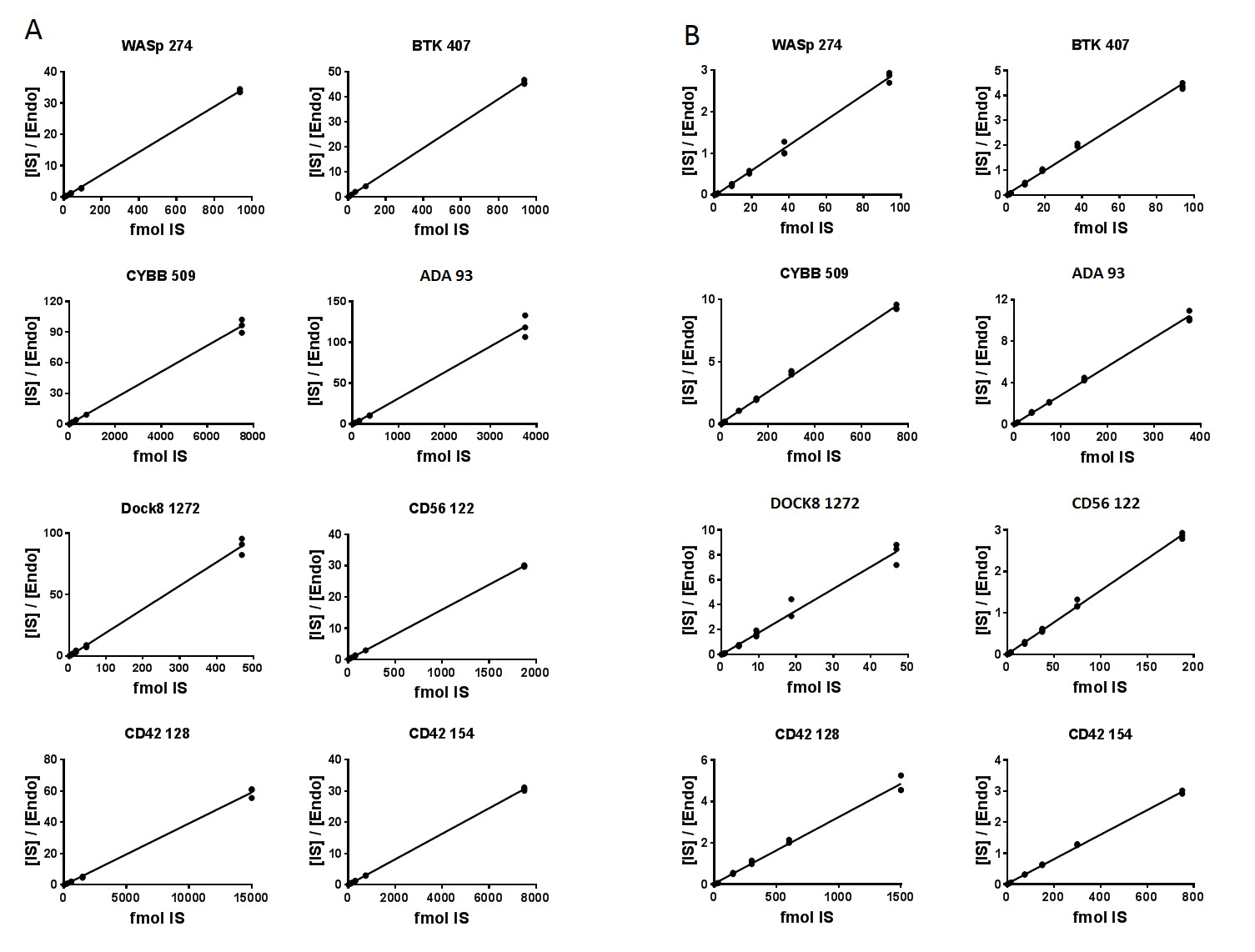

Supplement: Figure S3 — Linearity curves for each signature peptide. High fmol range plots (A) represent the full analyzed concentration range. Low fmol range plots (B) represent enlarged low concentration regions from A plots. [file Image_3.jpg]

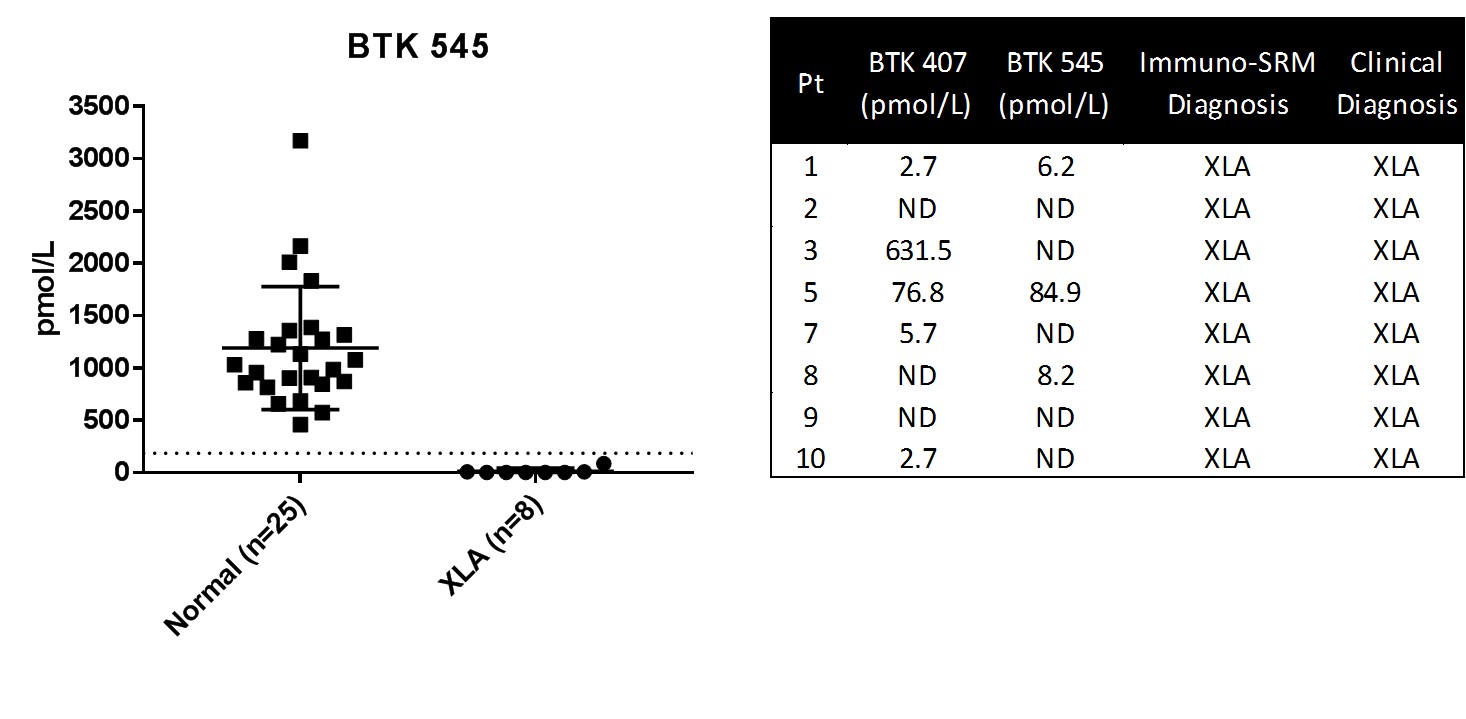

Supplement: Figure S4 — Analysis of XLA patients with secondary signature peptide BTK 545. ND, Not Detectable. Diagnostic cutoff was set at 1.75 SD below the normal mean. [file Image_4.JPEG]

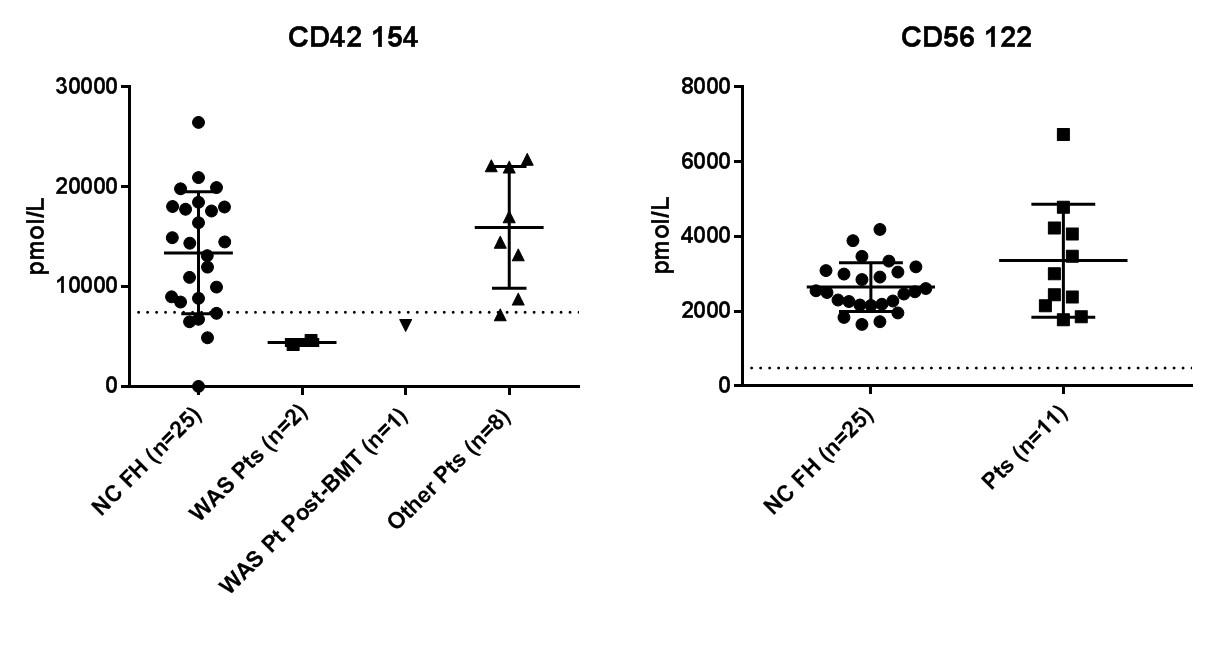

Supplement: Figure S5 — Normal Control and patient DBS peptide concentrations generated at FHCRC for secondary markers CD56 122 and CD42 154. [file Image_5.JPEG]

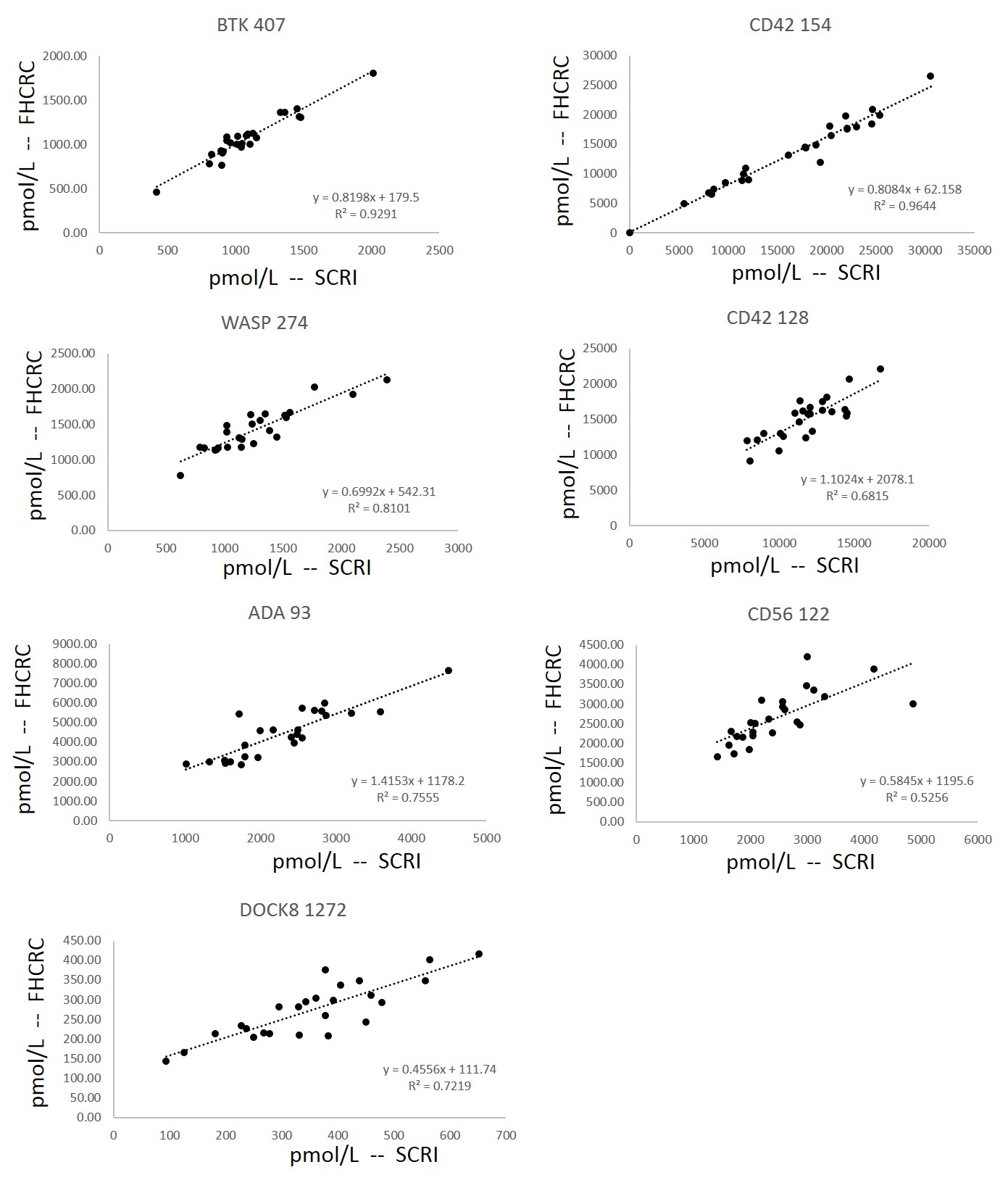

Supplement: Figure S6 — Correlation plots for measured peptide concentrations between Fred Hutchinson Cancer Research Institute (FHCRC) and Seattle Children's Research Institute. [file Image_6.JPEG]
