# Supplementary material for: Multiplexed Proteomic Analysis for Diagnosis and Screening of Five Primary Immunodeficiency Disorders From Dried Blood Spots
Source: Front Immunol. 2020 Apr 1;11:464. doi: 10.3389/fimmu.2020.00464 (PMC7141245; doi:10.3389/fimmu.2020.00464)
Supplement: Table S1 — Stock and final internal peptide concentrations and monoclonal antibody bead mass per sample for each target peptide. [file Table_1.pdf]

	500x Stock Concentration (nM)	Optimized Final Concentration (nM)	mAb Bead Mass (µg/sample)
WAS 274	625	1.25	4
BTK 407	625	1.25	1
CYBB 509	5000	10	0.1
ADA 93	2500	5	0.25
DOCK8 1272	312.5	0.625	2
CD42 128	10000	20	1
CD42 154	5000	10	0.5
CD56 122	1250	2.5	2
