# Supplementary material for: Multiplexed Proteomic Analysis for Diagnosis and Screening of Five Primary Immunodeficiency Disorders From Dried Blood Spots
Source: Front Immunol. 2020 Apr 1;11:464. doi: 10.3389/fimmu.2020.00464 (PMC7141245; doi:10.3389/fimmu.2020.00464)
Supplement: Table S2 — Peptide concentration data for patients measured at FHCRC. [file Table_2.pdf]

Pt	WASP 274 (pmol/L)	BTK 407 (pmol/L)	ADA 93 (pmol/L)	DOCK8 1272 (pmol/L)	CD42 128 (pmol/L)	CD42 154 (pmol/L)	CD56 122 (pmol/L)	Diagnosis	Notes
6	1668.6	21.0	2597.0	240.3	11906.4	22763.1	1778.8	XLA	
7	1541.6	17.7	3573.6	256.3	15071.2	17014.1	2383.3	XLA	
8	1736.6	5.3	3244.4	344.2	10077.6	13184.5	3006.1	XLA	
9	1977.8	3.0	4093.2	406.5	18928.4	22104.7	4781.6	XLA	
10	1667.8	21.6	2614.2	303.7	15832.6	21988.7	2151.0	XLA	
18	168.1	352.0	7060.1	179.2	3186.2	4207.0	1853.8	WAS	
19	41.9	692.1	4669.1	430.3	3633.9	4624.6	3470.8	WAS	
20-B	1044.6	1157.7	6130.4	310.9	10582.7	6115.7	6730.0	Normal	Post-BMT
23	1273.2	956.4	3900.2	258.4	13976.7	7180.6	4227.7	Normal	XL-CGD Pt: CYBB 509 Not Measured
25	790.5	583.1	2931.9	12.5	6993.2	8774.2	2446.0	DOCK8 Def.	
28	1204.5	919.6	105.8	230.3	12432.1	14458.2	4070.6	ADA Def.	PEG-ADA ERT
