# Supplementary material for: Multiplexed Proteomic Analysis for Diagnosis and Screening of Five Primary Immunodeficiency Disorders From Dried Blood Spots
Source: Front Immunol. 2020 Apr 1;11:464. doi: 10.3389/fimmu.2020.00464 (PMC7141245; doi:10.3389/fimmu.2020.00464)
Supplement: Table S3 — High-throughput gradient transitions for signature peptides. Primary markers are used for direct diagnosis of the specific associated PIDD. Secondary markers provide information related to markers of hematopoiesis associated targets. [file Table_3.pdf]

Disease Or Cell Target	Marker Type	Protein	Peptide	Sequence	Mass (Da)	Parent Ion (m/z)	Fragment Ion (m/z)
Wiskott-Aldrich Syndrome	Primary	WASP	WASP 274 - 288	AGISEAQLTDAETSK	1521.76	760.88 ++	[y10] - 1063.5266+, [y8] - 864.4309+, [y7] - 751.3468+, [y3] - 335.1925+, [b3] - 242.1499+
X-Linked Agammaglobulinemia	Primary	BTK	BTK 407 - 417	ELGTGQFGVVK	1135.63	567.81 ++	[y9] - 892.4887+, [y7] - 734.4196+, [y9] - 446.7480++
X-Linked Chronic Granulomatous Disease	Primary	CYBB	CYBB 509 - 521	TLYGRPNWDNEFK	1639.767	547.2670 +++	[y12] - 769.8730++, [y11] - 713.3309++, [y10] - 631.7993++
Adenosine Deaminase Deficiency	Primary	ADA	ADA 93 - 101	EGVVYVEVR	1049.173	525.2849 ++	[y6] - 764.4301+, [y5] - 665.3617+, [y4] - 502.2984+
DOCK8 Deficiency	Primary	DOCK8	DOCK8 1272 - 1283	TSGIVLSSLPYK	1264.466	632.8610 ++	[y10] - 1076.6350+, [y8] - 906.5295+, [y7] - 807.4611+
Platelets	Secondary	CD42	CD42 128 - 137	LTSLPLGALR	1040.254	520.8268 ++	[y8] - 826.5145+ , [y6] - 626.3984+ , [y4] - 416.2616+
	Secondary	CD42	CD42 154 - 165	TLPPGLLTPTPK	1234.483	617.8739 ++	[y10] - 1020.6088+ , [y10] - 510.8080++, [y9] - 462.2817++
NK Cells	Secondary	CD56	CD56 122 - 130	NAPTPQEFR	1059.128	530.2645 ++	[y7] - 874.4417+ , [y6] - 777.3890+ , [y5] - 676.3413+, [y4] - 579.2885+, [y7] - 437.7245++
